# A novel molecular imaging probe [^99m^Tc]Tc-HYNIC-FAPI targeting cancer-associated fibroblasts

**DOI:** 10.1038/s41598-023-30806-6

**Published:** 2023-03-06

**Authors:** Yanghongyan Jiang, Yaxin Tian, Bei Feng, Tingting Zhao, Liang Du, Xiaodong Yu, Qian Zhao

**Affiliations:** 1 Department of Nuclear Medicine, General Hospital of Ningxia Medical University, 804 Shengli St, Yinchuan, 750004 China; 2 Graduate School of Ningxia Medical University, Yinchuan, 750004 China

**Keywords:** Cancer imaging, Cancer imaging

## Abstract

Fibroblast activation protein (FAP) is higher expressed on cancer-associated fibroblasts (CAFs) in most malignant epithelial neoplasms, which is lower expressed in normal tissues. As a promising small molecular probe, FAP inhibitor (FAPI) shows the specific binding to FAP. This study aimed to explore a novel molecular probe [^99m^Tc]Tc-HYNIC-FAPI targeting CAFs. The in vitro characteristics of the probe were also evaluated. The FAPI targeting FAP was designed, synthesized and conjugated with the chelator 6-hydrazinylnicotinic acid (HYNIC) for radiolabeling with ^99m^Tc. The radiolabeling yield, radiochemical purity and stability were evaluated by Instant thin-layer chromatography (ITLC) and High performance liquid chromatography (HPLC). Lipophilicity was performed by the distribution coefficient test. The binding and migration ability of the probe was assessed using the FAP transfected tumor cell line. The radiolabeling yield of [^99m^Tc]Tc-HYNIC-FAPI was (97.29 ± 0.46) %. The radiochemical purity was more than 90% and kept stable until 6 h. The radioligand was shown as lower lipophilicity, of which logD7.4 value was − 2.38 $\pm$ 0.13. In vitro experiments, the results indicated that the probe showed binding properties, and inhibited the migration of tumor cells. The novel [^99m^Tc]Tc-HYNIC-FAPI probe was successfully radiosynthesized and exhibited good radiochemical purity, stability and in vitro binding ability to tumor cells. The [^99m^Tc]Tc-HYNIC-FAPI will be a promising SPECT/CT imaging probe.

## Introduction

Tumor cells and extracellular matrix components are collectively called the tumor microenvironment (TME). Cancer-associated fibroblasts (CAFs) are the most essential components of TME, which play a crucial role in tumor formation and development^1^, especially in relation to tumor angiogenesis, progression and metastasis^2,3^. Fibroblast activation protein (FAP) is hardly expressed in normal tissues and benign epithelial tumors, while it is overexpressed on CAFs in approximately 90% of malignant epithelial neoplasms^4^, such as gastric cancer, breast cancer, pancreatic cancer, colorectal cancer, etc^5–8^. Therefore, FAP is a new promising molecular target for the diagnosis of cancers. FAP inhibitor (FAPI) is a small molecular inhibitor, which specifically binding to FAP. FAPI was initially developed as an anticancer drug^9^, subsequently designed as a new class of radiopharmaceuticals^8,10^. Some molecular probes labeled with FAPI were reported as an exciting probe on tumor imaging.

Recently, the most reported FAPI imaging was focused on PET/CT imaging with ^68^Ga in preclinical and clinical studies^10–12^. They showed definite imaging characteristics. A promising ^68^Ga-labeled FAPI-04 radiotracer was presented with remarkably high uptake and good image contrast on PET/CT in 28 different kinds of cancers^12^. However, the PET/CT is not widely equipped, and the cost of positron emission radiotracer is expensive, as well as hard to prepare, which partly limits the application of FAPI imaging.

SPECT/CT with technetium-99 m (^99m^Tc) radiolabeled probes is one of the most widely used molecular imaging techniques. There were only a few studies on ^99m^Tc-labeled FAPI radiotracers. For example, [^99m^Tc]Tc-FAPI-34 was screened from several FAPI derivatives^13^, and [^99m^Tc]Tc-iFAP was designed based on the boroPro derivatives^14^. Therefore, more researches are still needed to clarify the efficacy of FAPI imaging with SPECT/CT.

**Figure 1:**
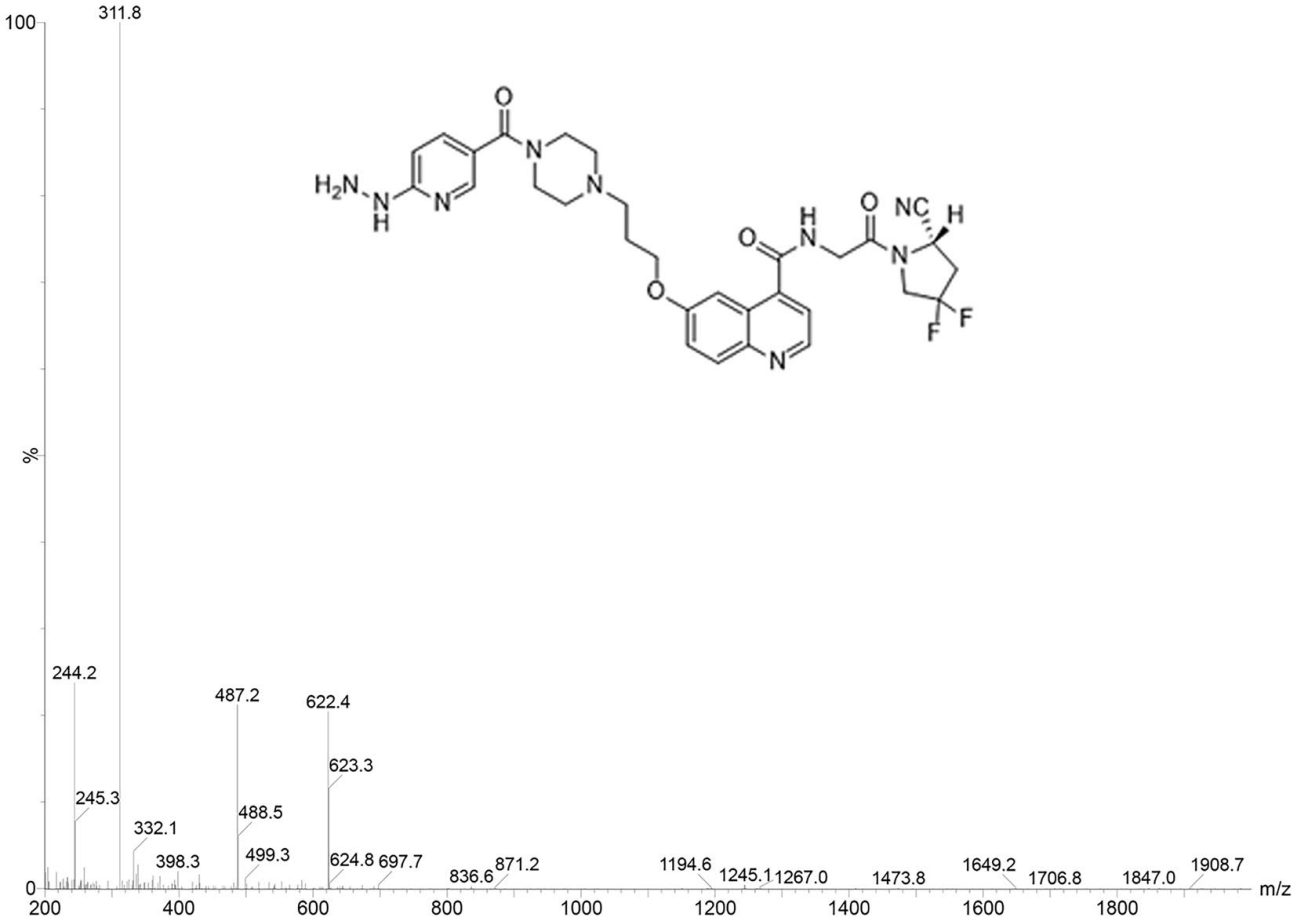
The structure and MS data of HYNIC-FAPI.

The goal of this study was to explore a new molecular probe [^99m^Tc]Tc- HYNIC-FAPI, and the in vitro characteristics were also evaluated (Fig. 1).

## Results

### Synthesis and radiolabeling of [^99m^Tc]Tc-HYNIC-FAPI

**Figure 2:**
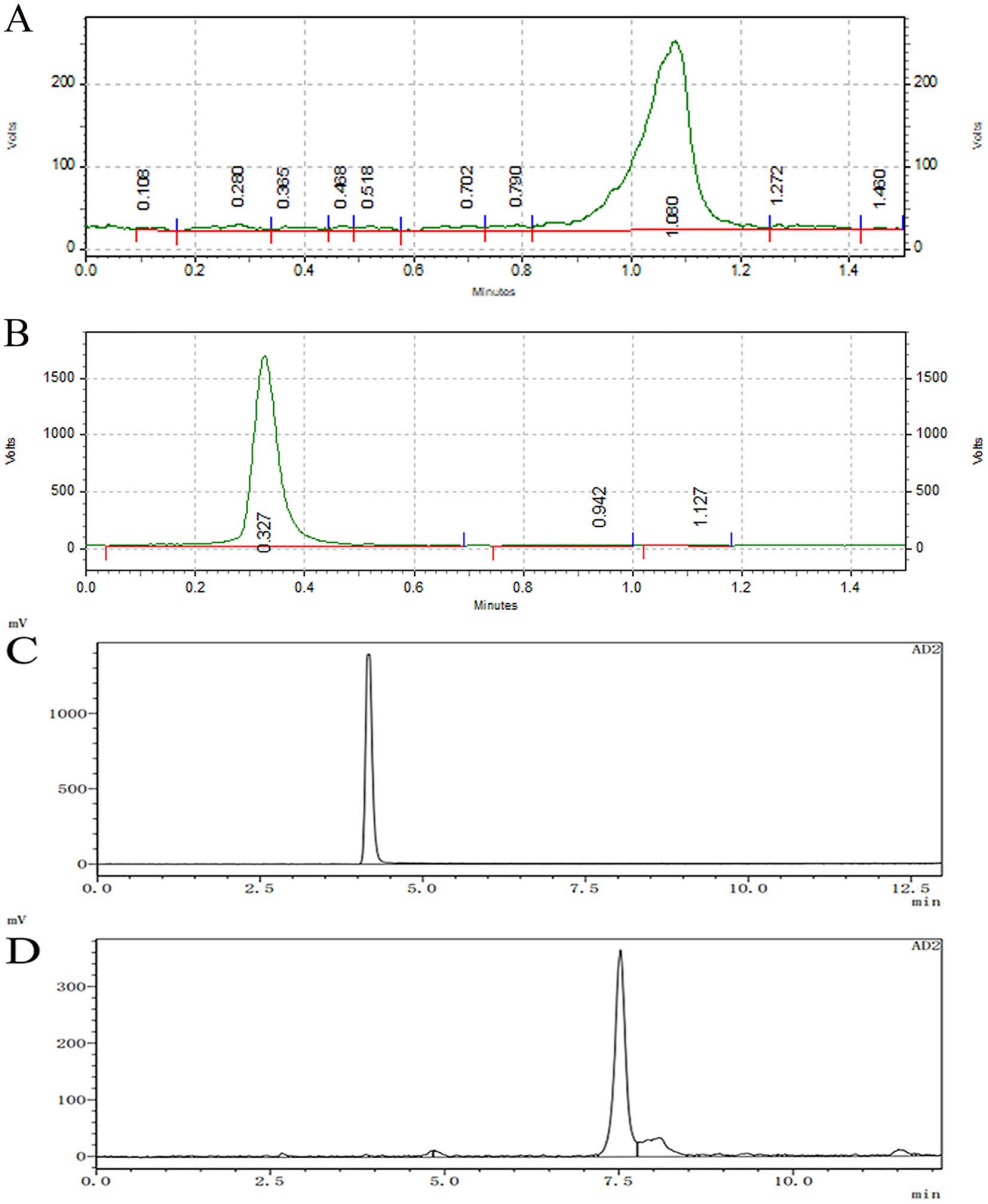
Identification of the probe by ITLC and HPLC. Retention time of ^99m^Tc (**A**) and [^99m^Tc]Tc-HYNIC-FAPI (**B**) by ITLC. HPLC pattern of ^99m^Tc (**C**) and [^99m^Tc]Tc-HYNIC-FAPI (**D**). The X-axis showed the running time during the process of testing.

The radiolabeling yield of [^99m^Tc]Tc-HYNIC-FAPI was (97.29 ± 0.46) % (Fig. 2B). The radiochemical purity of [^99m^Tc]Tc-HYNIC-FAPI was more than 90% by using HPLC (Fig. 2D). The ITLC and HPLC results of ^99m^Tc were shown in Fig. 2A and C.

### Stability and lipophilicity study

**Figure 3:**
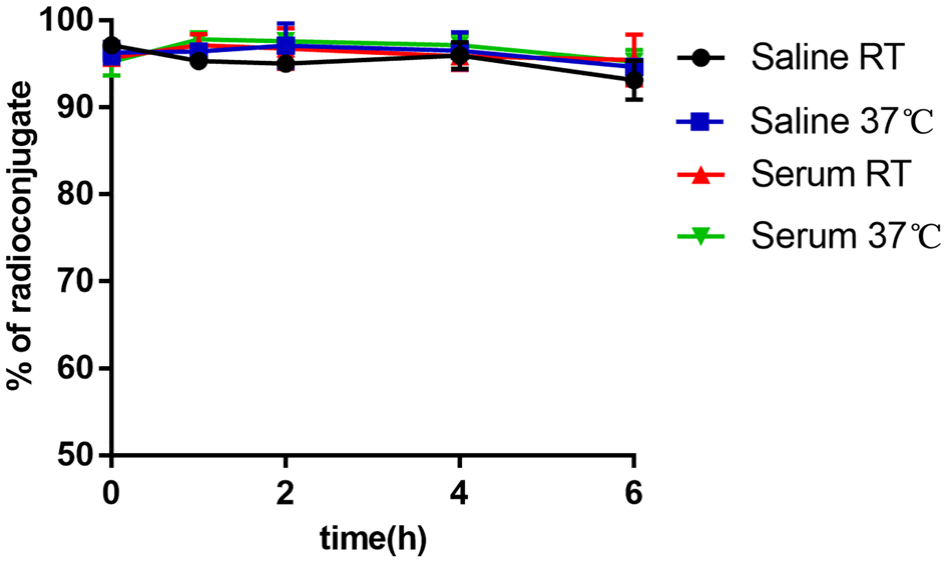
In vitro stability. The radiolabeling yield of [^99m^Tc]Tc-HYNIC-FAPI was incubated in saline and fetal bovine serum during 6 h, at room temperature (RT) and 37 °C.

The radiolabeling yield of [^99m^Tc]Tc-HYNIC-FAPI was stable in saline and serum until 6 h, which was still more than 90% (Fig. 3). There was no significant difference after incubation in saline or fetal bovine serum at room temperature and 37 °C for 6 h. These results showed a good in vitro stability of [^99m^Tc]Tc-HYNIC-FAPI.

The distribution coefficient (logD7.4) value was recorded as − 2.38 $\pm$ 0.13, which indicated a good hydrophilic performance.

### In vitro characteristics of [^99m^Tc]Tc-HYNIC-FAPI

#### Western blot

FAP transfected cell-line MCF-7-FAP was employed in cellular experiments because of the limited expression of FAPI in tumor cells. GAPDH was used as a standard control.

**Figure 4:**
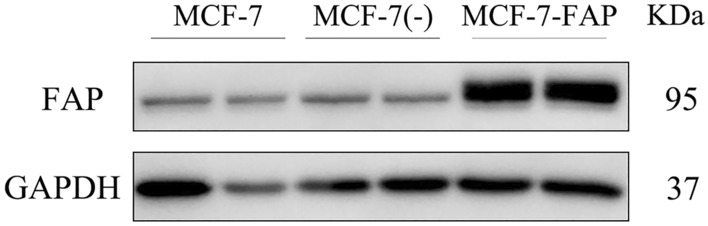
Western blotting results. It showed a successful expression of FAP in MCF-7 cell lines. (MCF-7(−), the MCF-7 cells transfected with empty vectors) (Full-length blots are presented in Supplementary Fig. 1).

The transfection was successful according to the Western blotting assay (Fig. 4). The results showed that FAP was overexpressed in MCF-7 cell line after transfection, while there was lower expression in the non-transfected cells as well as the empty vector control.

#### Cellular uptake

**Figure 5:**
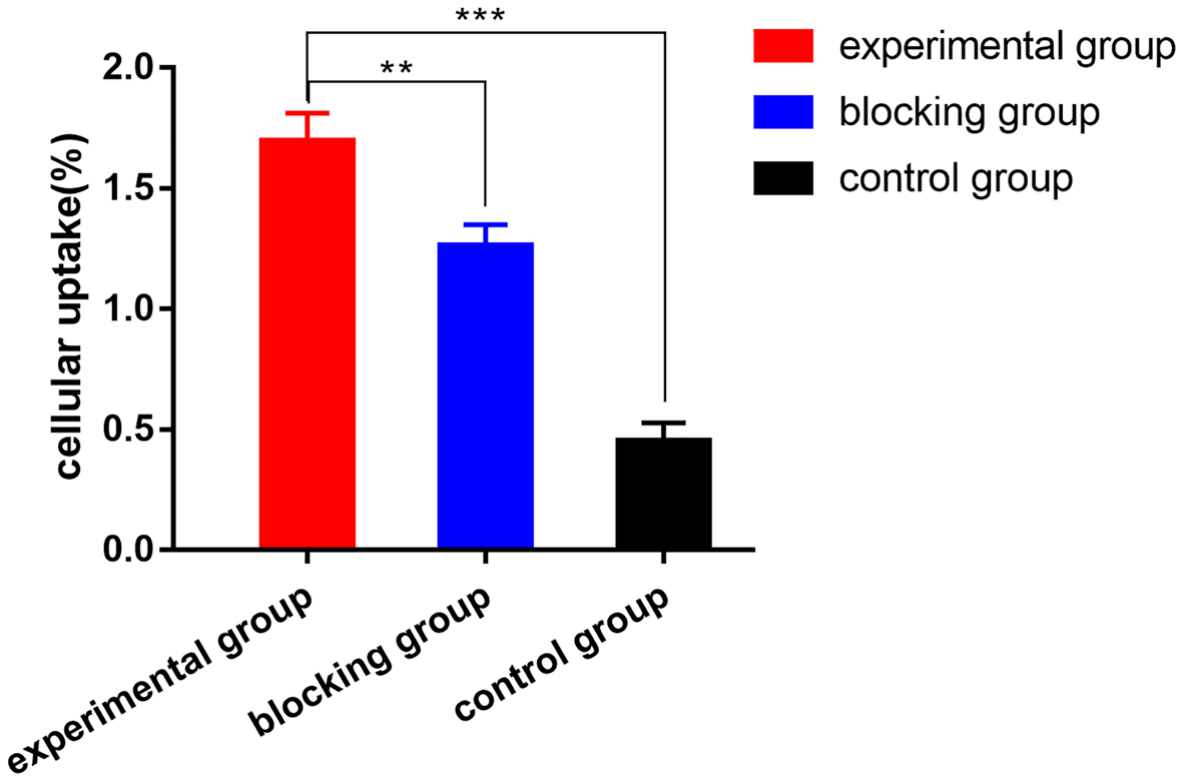
The in vitro cellular uptake rates of experimental, blocking and control groups at 3 h. * Statistical significance, *P* < 0.05.

The uptake rate of [^99m^Tc]Tc-HYNIC-FAPI was (1.69 ± 0.12) % after 3 h, while the blocking and control group were shown even poorly binding rate (Fig. 5).

#### Cell wound scratch assay

**Figure 6:**
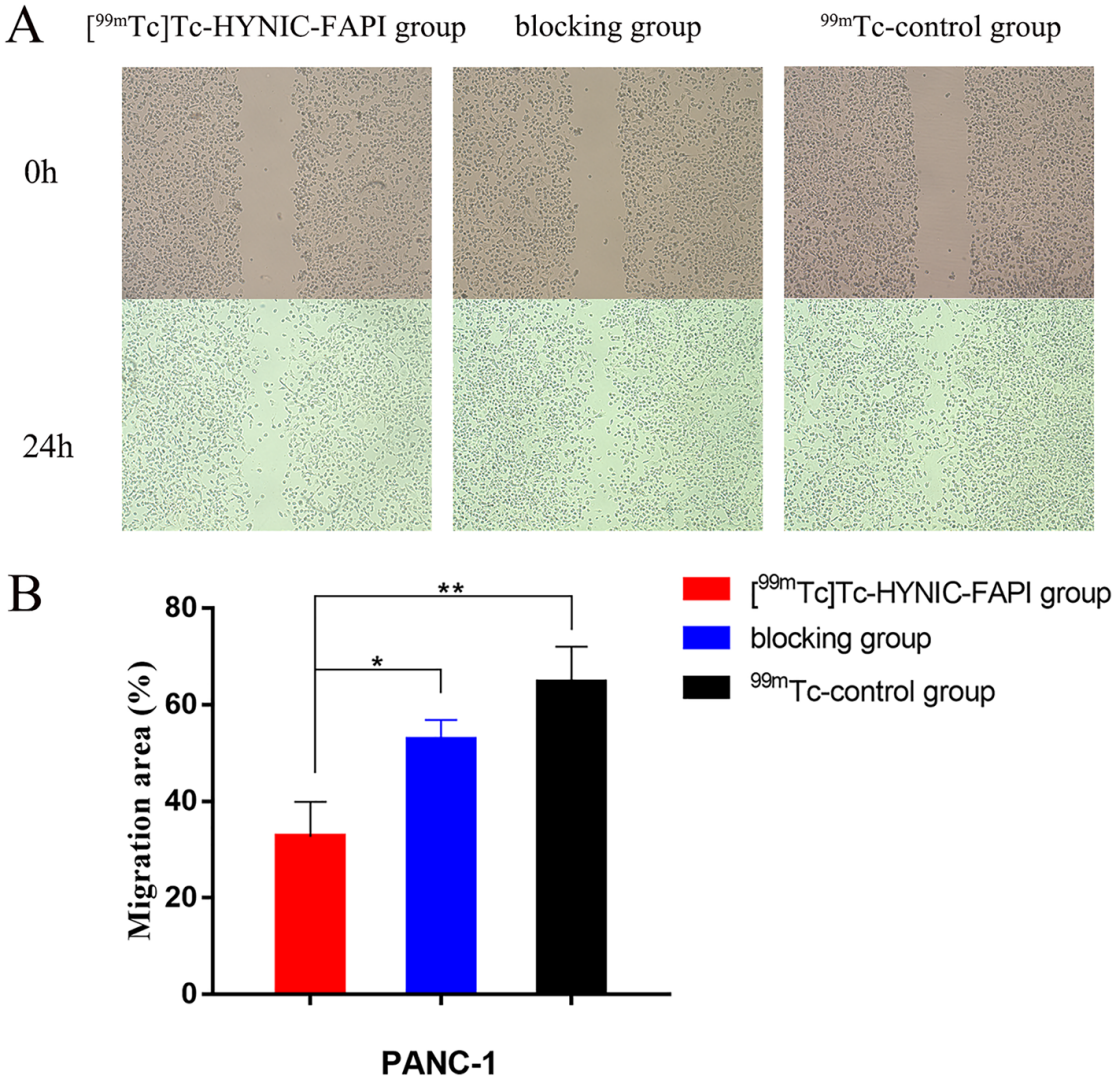
The effect of [^99m^Tc]Tc-HYNIC-FAPI on the migration of PANC-1 cells. (**A**) Scratch assay on PANC-1 cells showed significantly inhibited wound closure in cells incubated with [^99m^Tc]Tc-HYNIC-FAPI after 24 h. (**B**) Statistical histogram of scratch assay. * Statistical significance, *P* < 0.05.

Cell scratch wound assay was applied to observe the results of [^99m^Tc]Tc-HYNIC-FAPI on the migration of PANC-1 cells. The migration results were better shown in pancreatic cancer cells. As illustrated in Fig. 6, the migratory effect of PANC-1 cells was attenuated in the [^99m^Tc]Tc-HYNIC-FAPI group. The motility of PANC-1 cells of the [^99m^Tc]Tc-HYNIC-FAPI group was significantly different compared with the blocking and ^99m^Tc-control group (*P* < 0.05). All these data suggested that the proliferation of tumor cells was inhibited by [^99m^Tc]Tc-HYNIC-FAPI.

## Discussion

FAP is a type II transmembrane serine protein that shows higher expression in CAFs, which is hardly found in normal fibroblasts. FAP is shown both dipeptidyl peptidase and endopeptidase activities^15^. Due to its specific expression pattern, FAP is emerging as a promising target for diagnosis and therapeutic intervention. And FAPI is a unique molecular probe in tumor imaging, which can be used in tumor diagnosis, staging, therapeutic and prognostic assessment. In this study, we synthesized a new molecular probe [^99m^Tc]Tc-HYNIC-FAPI and explored its in vitro characteristics. Our main finding was that [^99m^Tc]Tc-HYNIC-FAPI can be radiolabeled and showed promising performance in tumor imaging.

^68^Ga or ^18^F radiolabeled with FAPI is designed as diagnostic radiopharmaceuticals for PET imaging, which developed and applied in clinical studies nowadays, such as [^68^Ga]Ga-FAPIs-02, 04, and 46. Previous studies have confirmed that the novel FAP-targeted tracers labeled with ^68^Ga demonstrated a higher sensitivity and tumor-to-nontumor contrast ratios than those attained with 2-[^18^F]F-FDG PET/CT in several different types of malignant tumors^11,16,17^. The higher contrast and lower radiation of 2-[^18^F]F-FAPI-74 PET/CT imaging make it possible to produce with large-scale and to be used in multiple clinical applications^18^. Based on the successful application of FAPI probes radiolabeled with ^68^Ga or ^18^F, a new class of radiopharmaceuticals labeled with ^99m^Tc was designed and evaluated.

We successfully radiolabeled [^99m^Tc]Tc-HYNIC-FAPI probe. The chelator 6-hydrazinylnicotinic acid (HYNIC) was employed for linking of the FAP-targeting site to ^99m^Tc isotopes. It is also well-known that HYNIC is a bifunctional coupling agent with two nitrogen atoms of the hydrazine group, and the advantage of the ternary ligand system was higher radiolabeling yield, good stability and hydrophilicity^19^.The HYNIC-(tricine/TPPTS) complex has been reported successfully used for radiolabeling^20,21^. In the present study, the labeling rate of [^99m^Tc]Tc-HYNIC-FAPI was (97.29 ± 0.46) %, which was paralleled with other studies. Among them, one study synthesized [^99m^Tc]Tc-iFAP, in which HYNIC and two EDDA molecules was used as co-ligands leading to a high in vitro and in vivo stability^14^. The radioconjugate exhibited excellent hydrophilicity, which is characterized by lower systemic toxicity and rapid clearance through the urinary system in further in vivo experiments.

FAP-transfected cancer cells were used to evaluate the uptake ability of [^99m^Tc]Tc-HYNIC-FAPI because of the lower or non-expression of FAP protein in tumor cells. We also confirmed the different level of FAP expression in MCF-7-FAP and MCF-7 by the Western blot assay. According to the results of cellular uptake assay, the increased uptake of [^99m^Tc]Tc-HYNIC-FAPI was observed in MCF-7-FAP cells when compared to the group blocking with HYNIC-FAPI and control group.

The cell scratch wound assay and PANC-1 cell line were used for migration test. When applied with [^99m^Tc]Tc-HYNIC-FAPI, the migration of PANC-1 cells were significantly inhibited. It was reported that FAP was highly expressed in human pancreatic ductal adenocarcinoma (PDAC) cells^22^. Over-expression of FAP might bind the FAK protein that reduces its phosphorylation, which thus results in decreasing migration ability of MCF-7 cells^23^. Consistent with our findings, when applied with [^99m^Tc]Tc-HYNIC-FAPI in cell scratch wound assay, the migration of MCF-7-FAP cells were less attenuated in each group within 24 h (The motility of MCF-7-FAP cells applied with the [^99m^Tc]Tc-HYNIC-FAPI is presented in Supplementary Fig. 2).

Although we confirmed the better performance of our novel molecular imaging probe [^99m^Tc]Tc-HYNIC-FAPI, a further imaging study on different types of malignant tumor-beared mice was still needed.

## Conclusions

This study demonstrated that [^99m^Tc]Tc-HYNIC-FAPI can be successfully radiosynthesized. The [^99m^Tc]Tc-HYNIC-FAPI probe exhibited good in vitro stability and targeting ability, and inhibited the migration ability of tumor cells. [^99m^Tc]Tc-HYNIC-FAPI is a promising small molecular probe for SPECT/CT tumor imaging.

## Materials and methods

### Synthesis and radiolabeling

The HYNIC-FAPI probe was chemically modified and synthesized from Nanchang Tanzhen Biotechnologies Co., Ltd. (Jiangxi, China). The purity of HYNIC-FAPI was 98%, and the structure of the product was identified by MS (Fig. 1). ^99m^Tc is obtained from the ^99^Mo-^99m^Tc generator supplied by China Atomic Energy Research Institute (Beijing, China). 10 mg tricine (Tricine buffer, free acid) and 20 mg TPPTS (Tris(3-sulfonatophenyl) phosphine, sodium salt) dissolved in 5 ml saline (95%), then 50 μl HYNIC‑FAPI (1 μg/μl), 50 μl ^99m^T (185 MBq) and 25 μl fresh SnCl_2_·2H_2_O(1 mg/ml in 0.2 M HCL) were added into 200 μl mixture above in turn. All the reagents were mixed and heated at 100 °C for 30 min to synthesize [^99m^Tc]Tc-HYNIC-FAPI.

### Quality control

The radiolabeling yield and radiochemical purity of [^99m^Tc]Tc-HYNIC-FAPI were checked by Instant thin-layer chromatography (ITLC) and High Performance Liquid Chromatography (HPLC) technique.

For ITLC, Whatman No. 1 filter paper was used as stationary phase and acetonitrile as mobile phases. The mixture was spotted at the baseline of the Whatman No. 1 filter paper. The retention time and the %RCY was calculated automatically by the Mini Scan TLC device (BIOSCAN Co., Ltd. (America)).

HPLC was determined by C18 column (4.6 × 250 mm, 5 μm) under working conditions maintained with a flow rate of 1 ml/min in 0.1% TFA/water (A) and 0.1% TFA/acetonitrile (B). The injection volume was 20 μl and the detection time was set as 20 min (12–32% B).

### Stability and lipophilicity study

ITLC was also carried out for the assessment of [^99m^Tc]Tc-HYNIC-FAPI in vitro stability at 0, 2, 4, 6 h. [^99m^Tc]Tc-HYNIC-FAPI was incubated in saline and fetal bovine serum separately at room temperature and 37 °C.

The lipophilicity of the radioconjugate was evaluated by distribution coefficient (logD7.4). Approximately 100 μl 1.85 MBq [^99m^Tc]Tc-HYNIC-FAPI was added in a test tube including 1 ml phosphate buffer saline (PBS, pH = 7.4) and 1 ml n-octanol. The complex was fully mixed on a Vortex mixer for 5 min. After centrifugation at 5000 rpm for 5 min, 100 μl solution of the both two layers was collected separately. The radioactive counts were measured using a well-type gamma detector. The formula of calculation of logD7.4 was used as follow:

$${\text{logD}}\, = \,{\text{log }}\left( {{\text{activity concentration in n}} - {\text{octanol phase}}/{\text{ activity concentration in aqueous phase}}} \right). $$

### Cell culture and transfection

MCF-7 human breast cells were purchased from Procell Life Science & Technology CO., Ltd. (Wuhan, China). The cells were cultivated in Dulbecco's modified Eagle's medium (DMEM) containing 10% fetal bovine serum and 1% penicillin–streptomycin solution at 37 °C/5% carbon dioxide. Lentiviruses encoding with the human FAP-gene were obtained from Genechem Technology Co., Ltd. (Shanghai, China). All transfections were implemented according to the manufacturer’s instructions. The lentiviral vector was transiently transfected into MCF-7 cells. Stable cell lines were screened with puromycin.

### Western blot

Total protein was extracted using ice-cold whole cell lysis assay (KeyGEN BioTECH). After centrifugation at 14,000 × *g* for 15 min at 4 °C, the protein was quantified using the BCA protein assay kit (KeyGEN BioTECH). Then, the samples were separated on SDS-PAGE gels and transferred to PVDF membranes (Millipore) for 1.5 h. The membranes were blocked with 5% bovine serum albumin (BSA) for 1.5 h at room temperature. Next, the membranes were incubated with the anti-Fibroblast activation protein (1:1000, Abcam) overnight at 4 °C and subsequently washed three times by Tris Buffered Saline with Tween 20(TBST-1 ×). After incubation with the secondary antibody (1: 5000, Bioss) at room temperature for 1 h and washout for three times, the Bio-Rad Imager was employed to show the band of target proteins in detail.

### Cellular uptake

MCF-7-FAP and MCF-7 cells were incubated with 5 × 10^5^ cells/1 ml in 12-well plate overnight. The cells were divided into experimental, blocking, and control groups. MCF-7-FAP cells were used in experimental group, while MCF-7 cells in the control group. In blocking group, 1 μl unlabeled HYNIC-FAPI (1 μg/μl) was added into MCF-7-FAP cells 30 min earlier. 2 μl [^99m^Tc]Tc-HYNIC-FAPI (37 kBq) was added directly into the groups and then incubated for 3 h at 37 °C. All wells were washed three times with 500 μl PBS. Next, trypsin containing EDTA was used to lyse the cells, and the cells were washed thrice. Radioactivity was evaluated by Automatic Gamma Counter (Perkinmer). The radioactivity counts of cells and medium were measured, respectively.

### Cell wound scratch assay

PANC-1 cells were seeded in six-well plates. When the cell density reached 90%, the monolayer was scratched using a 200 μl plastic pipette tip, and the cells were washed with PBS in order to remove the floating cells. The PANC-1 cell experiment was divided into [^99m^Tc]Tc-HYNIC-FAPI, blocking, and ^99m^Tc-control groups. Images were acquired by inverted microscopy at 0 h and 24 h later. Calculation formula: Migration area (%) = (0 h scratch area − 24 h scratch area)/0 h scratch area $\times$ 100%.

### Statistical analysis

All results were presented as the mean ± standard deviation (SD). Comparison of the means of different groups was performed using Independent-sample t-test by GraphPad Prism 7. Statistical significance was defined as *P* < 0.05.

## Supplementary Information

Supplementary Information 1.

Supplementary Information 2.

## Data Availability

All data generated or analyzed during this study are included in this published article (and its Supplementary Information files).
