# Supplementary material for: A novel molecular imaging probe [99mTc]Tc-HYNIC-FAPI targeting cancer-associated fibroblasts
Source: Sci Rep. 2023 Mar 6;13:3700. doi: 10.1038/s41598-023-30806-6 (PMC9988823; doi:10.1038/s41598-023-30806-6)
Supplement: Supplementary file 1 — Supplementary Information 1. [file 41598_2023_30806_MOESM1_ESM.docx]

**Supplementary Fig. 1 Full-length blots**

**Western blot—FAP (95KD)、GAPDH (37KD)**


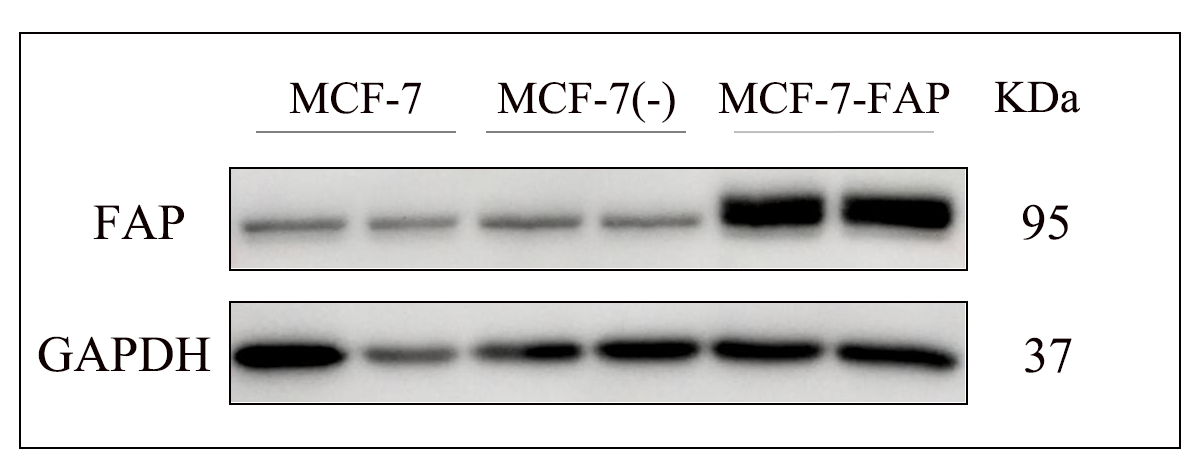


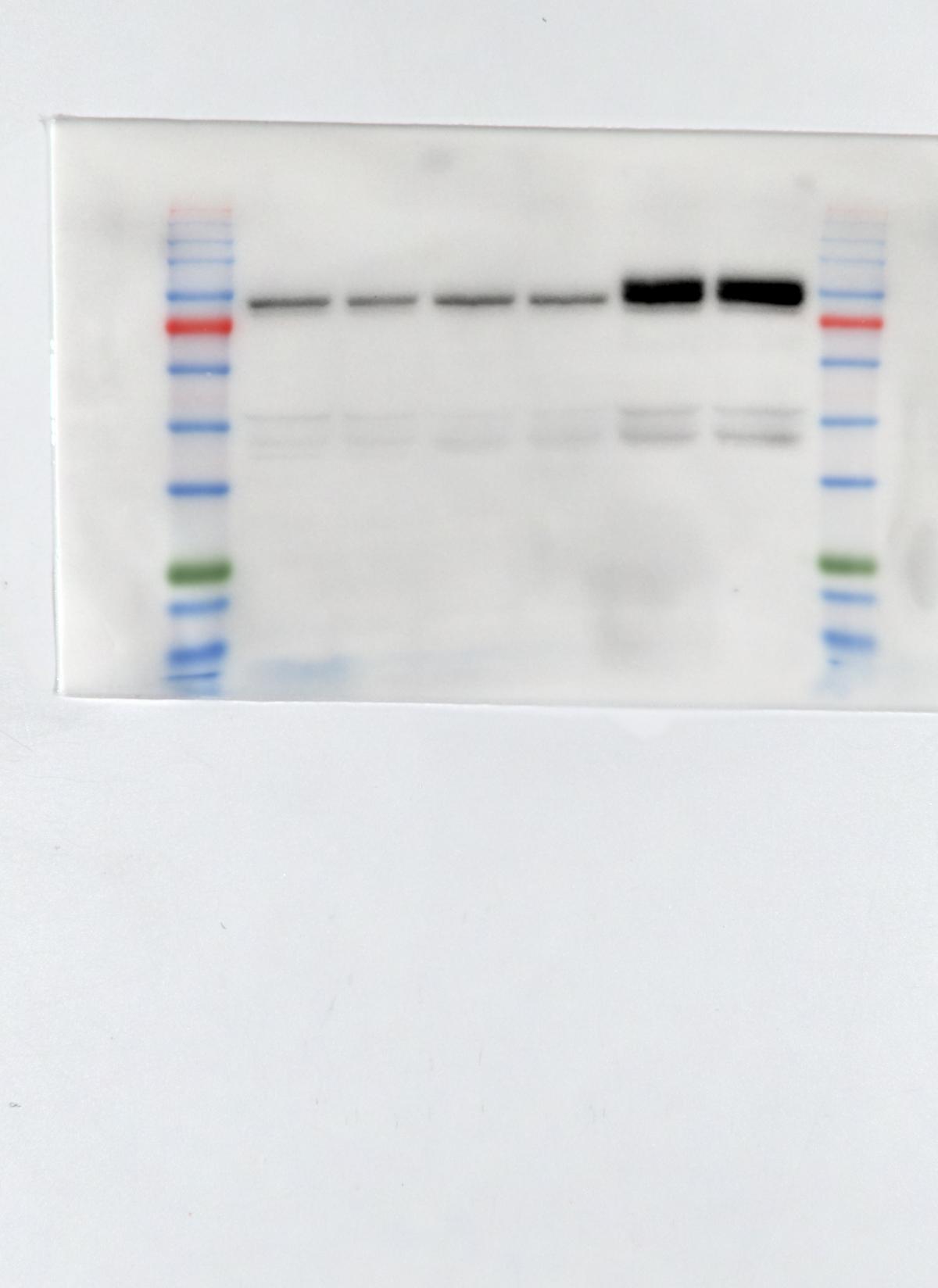


MCF-7-FAP

MCF-7(-)

MCF-7

60kDa

75kDa

45kDa

35kDa

25kDa

 the cropping of the blot

100kDa


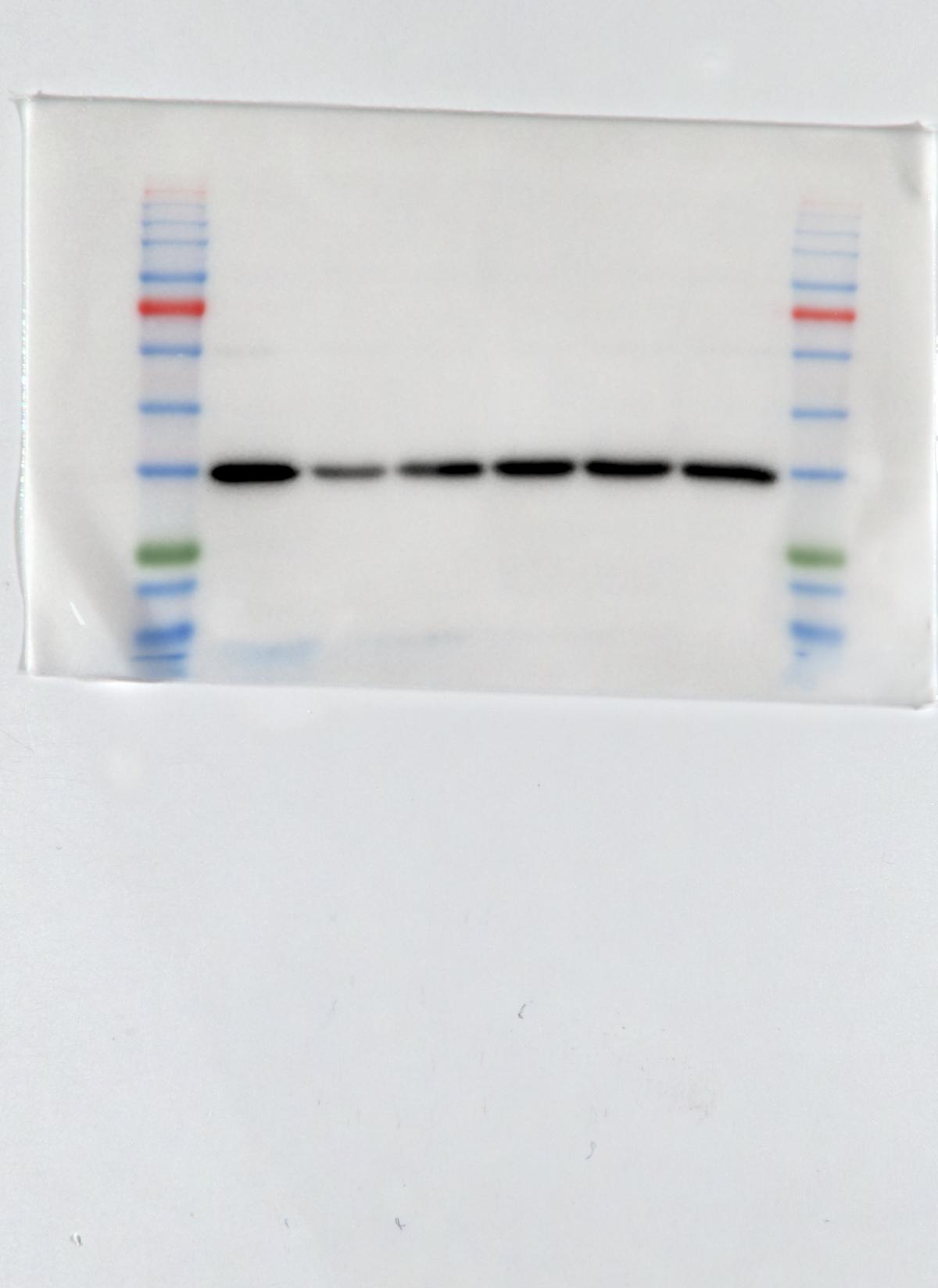


60kDa

45kDa

35kDa

75kDa

100kDa

25kDa

 the cropping of the blot
