# Supplementary material for: A novel molecular imaging probe [99mTc]Tc-HYNIC-FAPI targeting cancer-associated fibroblasts
Source: Sci Rep. 2023 Mar 6;13:3700. doi: 10.1038/s41598-023-30806-6 (PMC9988823; doi:10.1038/s41598-023-30806-6)
Supplement: Supplementary file 2 — Supplementary Information 2. [file 41598_2023_30806_MOESM2_ESM.docx]

**Supplementary Fig. 2**


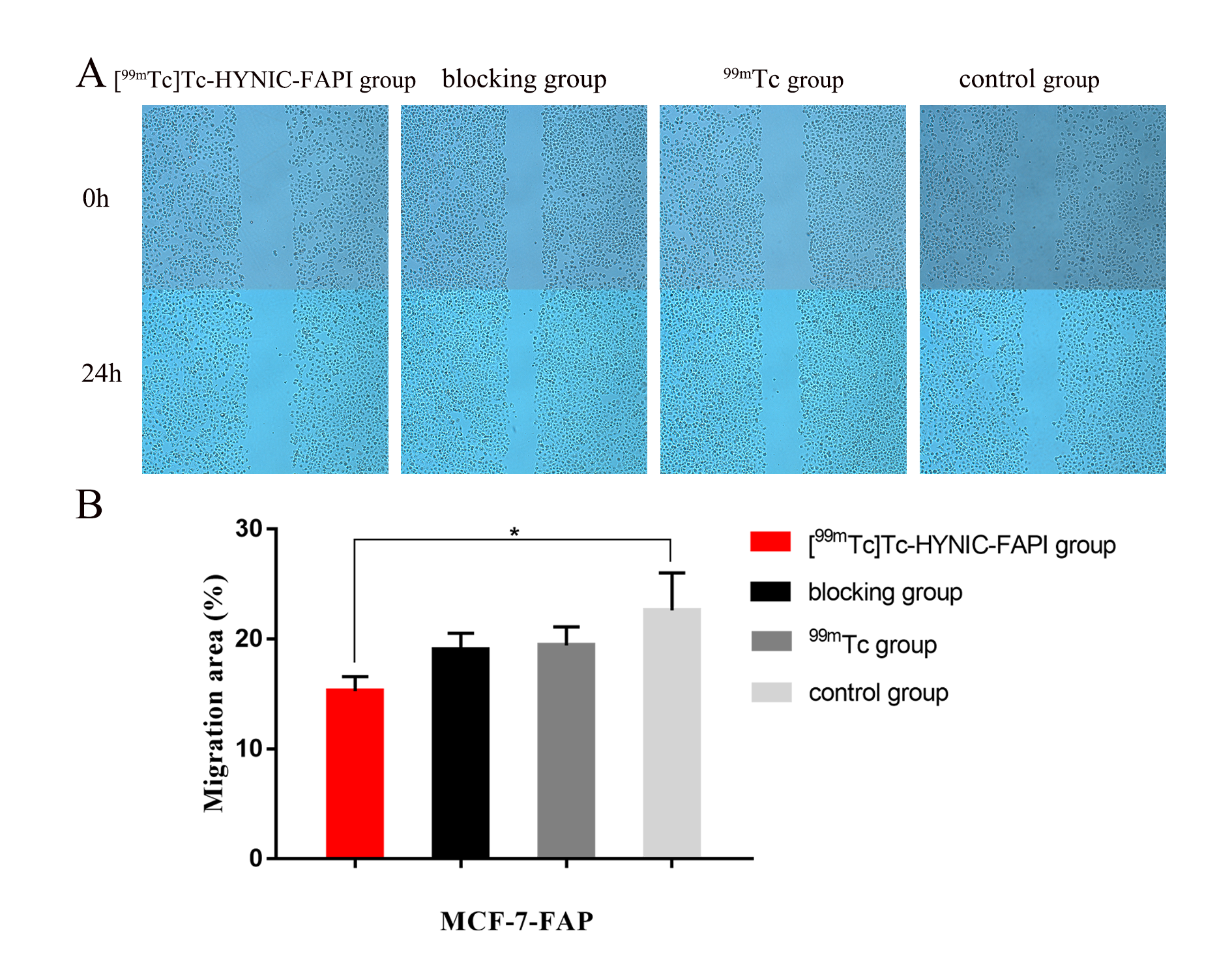


Supplementary Fig. 2：MCF-7-FAP Cell wound scratch assay.

As illustrated in Supplementary Fig. 2A, the migratory effect of MCF-7-FAP cells was attenuated in [^99m^Tc]Tc-HYNIC-FAPI group. The motility of MCF-7-FAP of the [^99m^Tc]Tc-HYNIC-FAPI and control groups was significantly different (*P*<0.05). However, the migration trend was not obvious in MCF-7-FAP cells.
